# Fluorescence Imaging Characterization of the Separation Process in a Monolithic Microfluidic Free-Flow Electrophoresis Device Fabricated Using Low-Temperature Co-Fired Ceramics

**DOI:** 10.3390/mi13071023

**Published:** 2022-06-28

**Authors:** Pedro Couceiro, Julián Alonso-Chamarro

**Affiliations:** Sensors & Biosensors Group, Department of Chemistry, Autonomous University of Barcelona, Edifici Cn, 08193 Bellaterra, Catalonia, Spain; pedro.couceiro@uab.es

**Keywords:** microfluidics, free-flow electrophoresis, fluorescence imaging, spatial-temporal pH characterization, low-temperature co-fired ceramics

## Abstract

A monolithic microfluidic free-flow electrophoresis device, fabricated using low-temperature co-fired ceramic technology, is presented. The device integrates gold electrodes and a 20 µm thick transparent ceramic optical window, suitable for fluorescence imaging, into a multilevel microfluidic chamber design. The microfluidic chamber consists of a 60 µm deep separation chamber and two, 50 µm deep electrode chambers separated by 10 µm deep side channel arrays. Fluorescence imaging was used for in-chip, spatial-temporal characterization of local pH variations in separation conditions as well as to characterize the separation process. The device allowed baseline resolution separation of a sample mixture of Fluorescein, Rhodamine 6G, and 4-Methylumbelliferone at pH 7.0, in only 6 s, using 378 V.s/cm. The results demonstrate the possibility of studying a chemical process using fluorescence imaging within the traditional fields of low-temperature co-fired ceramics technology, such as high-electrical-field applications, while using a simple fabrication procedure suitable for low-cost mass production.

## 1. Introduction

**Figure 1:**
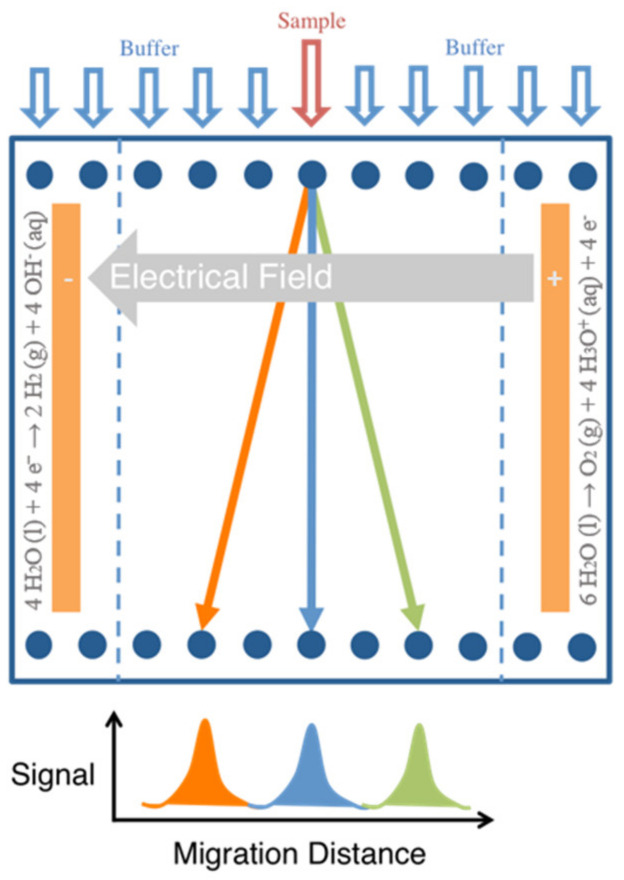
General principal of separation in free-flow electrophoresis, where a sample stream is continuously fed into the separation chamber and deflected laterally in an electric field based on the analyte’s electrophoretic mobilities.

Introduced in the late 1950s [1,2], free-flow electrophoresis (FFE) is a continuous separation technique, schematically described in Figure 1, in which a liquid sample mixture stream is continuously fed into a separation chamber where an electrical field is applied orthogonally to a liquid electrolyte flow direction. The sample mixture is, therefore, separated in space, allowing the collection of each sample mixture fraction in a different outlet. 

Benchtop FFE devices consist of two isolating plates separated by ion-exchange membranes which define the depth of the fluidic chambers and mechanically isolate the electrode chambers from the central separation chamber [3]. These physical barriers prevent gas bubbles, produced by water electrolysis, from entering the separation chamber, while ensuring electrical connectivity, between chambers. However, only moderate separation efficiency is obtained since the device’s large dimensions and volume limit joule heating dissipation even if the devices are placed in actively cooled plates, therefore limiting the applicable electrical field. Nonetheless, FFE has been used to separate a wide variety of compounds of different mass scales [3,4], such as cells, organelles, proteins, organic molecules mixtures [1], and nanoparticles [5].

Miniaturization of FFE devices [6,7,8,9] increases surface-to-volume ratio, and consequently joule heating dissipation, allowing the application of higher electric field, while decreasing the sample volume necessary for separation. Although promising, miniaturization of FFE has been technically challenging. High-sensitivity detection systems, such as fluorescence, must be implemented due to the optical path length reduction, while the increased importance of surface chemistry control at the microscale accentuates the need for the microfluidic platforms to be fabricated with the same substrate material. Ion exchange membranes have been integrated into microfluidic FFE (µFFE) devices [10,11,12,13,14,15], but have proven to be mechanically unstable at working flow rates and present limited stability in extreme pH conditions, requiring regular replacement. Alternatively, an extended number of strategies and alternative structures, which tries to mimic at the microscale the function of ion exchange membranes in benchtop FFE devices have been integrated into µFFE devices [16,17,18,19,20]. Most significantly, microstructures generating high hydrodynamic resistance regions [21,22,23,24,25,26], such as microchannel arrays or parallel shallow side banks, have been integrated into microfluidic devices fabricated in a wide variety of materials, such as glass, polymers, and multi-materials.

Low-temperature co-fired ceramics (LTCC) technology has emerged as an excellent substrate for the construction of such devices. Its multilayer approach, originally developed for the fabrication of multilayer flat circuits, allows complex 3D fluidic networks to be easily fabricated in the 10 cm to 10 μm range [27] in both open or embedded channel configurations and has been applied in the development of microfluidic platforms which integrate multiple unitary operations, such as sampling, mixing, filtration, and separation for analytical chemistry applications [28,29,30,31,32,33,34,35,36,37,38,39,40,41,42,43,44]. The significant historical limitation of using optical detection in LTCC monolithic devices due to the substrate opacity has recently been overcome by monolithic integration of an LTCC layer with thicknesses less than 50 μm, which, functioning as transparent optical windows, have allowed optical absorbance [45] and fluorescence imaging [27] to be used for in-chip chemical process characterization. The technology compatibility with conductive, dielectric, and resistor screen-printable pastes has allowed researchers to integrate microfluidic platforms with heating elements, electrochemical detection (amperometric, potentiometric, and conductiometric), as well as all the electronics necessary for signal acquisition and data processing [34,35,37,38] into devices fabricated using the same substrate material. Furthermore, the fabrication procedure ensures monolithic ceramic devices, with homogeneous surface chemistry as well as homogeneous physical properties, without requiring special facilities (clean rooms), enabling rapid, low cost, prototyping.

In this work, we present a monolithic µFFE device, with integrated transparent ceramic optical window and gold electrodes, fabricated using Low Temperature Co-Fired Ceramic (LTCC) technology. Due to the continuous flow nature of the FFE concept, a multilevel design, where closed electrode chambers are connected to the separation chamber by shallow side channels, was adopted. A mixture of fluorescence dyes were used in order to characterize the separation capabilities of the µFFE device as a function of separation power. Fluorescence imaging of 4-Methylumbelliferone (4MU) was studied for its potential use as a tool for spatial-temporal characterization of pH in separation conditions. The protocol proposed opens the possibility of spatial-temporal characterization of local pH in complex chemical processes, allowing comprehensive visual description of this chemical phenomenon using a simple and low-cost experimental setup. To our knowledge, this is the first time that a microfluidic platform with a monolithically integrated transparent optical window suitable for fluorescence imaging and gold electrodes suitable for high voltage applications has been fabricated using the same ceramic substrate. Moreover, the simple fabrication procedure presented is suitable for low-cost mass production.

## 2. Materials and Methods

### 2.1. Chemicals and Experimental Setup

All reagents were purchased from Sigma-Aldrich (Mollet del Vallès, Spain) All aqueous solutions were prepared in double distilled water. A stock solution of 5 g/L 4-Methylumbelliferone (4MU) was prepared in dimethyl sulfoxide (DMSO). Stock solutions of 3 g/L Rhodamine 6G (R6G) and 5 g/L Fluorescein (Fl) were prepared in MiliQ water. Buffer solution consisted of 10 mM 4-(2-hydroxyethyl)-1-piperazineethanesulfonic acid (HEPES) adjusted to pH 7.0. Working solutions of 0.25 mM 4MU, 0.25 mM Fl, 0.25 mM R6G, and 0.2% of (hydroxypropyl)methylcellulose (HPMC) in 10 mM HEPES, and of 0.25 mM 4MU, 0.2% of HPMC in 10 mM HEPES were prepared. All solutions were filtered through 0.22 μm membrane filters after preparation. In order to avoid air bubbles, the sample and buffer solutions were degassed in vacuum before flow operations.

In order to eliminate cross-contamination, the ceramic microfluidic platform was exposed to a sintering cycle before each experiment. The microfluidic device was mounted in an in-house built multichannel connector and connected to a 1 mL syringe (Hamilton Gastight 1000 TLL, Biogen Científica, Spain) and a 100 μL glass syringe (Hamilton Gastight 1710 TLLX, Biogen Científica, Spain) using Teflon tubes (0.8 mm i.d.) fitted with 1/4”-28 fluidic connectors (Idex p-335, VWR, Spain) and Luer-Lock-to-1/4”-28 adapters (Idex p-658, VWR, Spain). The syringes were mounted on syringe pumps (New Era Syringe Pumps NE-500, Biogen Científica, Spain). The microfluidic devices were connected to a programmable high voltage power supply (Labsmith HVS448-3000D, Mengel Engineering, Denmark). Fluorescence imaging of the microfluidic device was performed using a UV-A lamp (Philips PL-S 9W UV-A/2P 1CT) as a light source, and a CCD Camera (Nikon D90) as an optical detector. The CCD camera, the syringe pumps, and the high voltage power supply were controlled using a PC. Image processing and analysis was performed using ImageJ software. The excitation/emission spectra were obtained using of a spectrofluorometer (Fluorolog-1, Horiba, Kyoto, Japan).

### 2.2. Materials and Apparatus for the Construction of the Ceramic Microfluidic Device

DuPont 951 Green Tapes with 115 µm unfired thickness (DuPont 951 PT, CCI Eurolam, Spain) and 254 µm unfired thickness (DuPont 951 PX) were employed as ceramic substrates. The ablation of the ceramic substrates was performed using IR Nd:YAG laser equipment (LPKF Protolaser 200, Garbsen, Germany), at constant laser power (1.3 kW) and pulse frequency (40 KHz). Ceramic substrates were aligned in aluminium plates and laminated in a uniaxial hydraulic press (Talleres Francisco Camp S.A., Granollers, Spain) at 70 °C and 30 bars. Screen printing of DuPont 5742, DuPont QQ550 and DuPont 6146 pastes into ceramic substrates was performed using 125 mesh nylon screen-printing screens on a semi-automatic screen-printing machine (DEK 248 Vision, ASM, Barcelona Spain). Ceramic substrates sinterization was performed using a programmable box furnace (Carbolite CBCWF11/23P16, Afora, Spain), applying a temperature profile consisting of 5 stages. In the first stage, the temperature was increased from room temperature to 350 °C, at 10 °C min^−1^. A 30 min stabilization stage at 350 °C followed by a second heating ramp to 850 °C, at 10 °C min^−1^, was carried out. Finally, after 30 min of stabilization at 850 °C, the devices where cooled to room temperature.

## 3. Results and Discussion

### 3.1. Design and Fabrication of the Microfluidic Free Flow Electrophoresis Device

**Figure 2:**
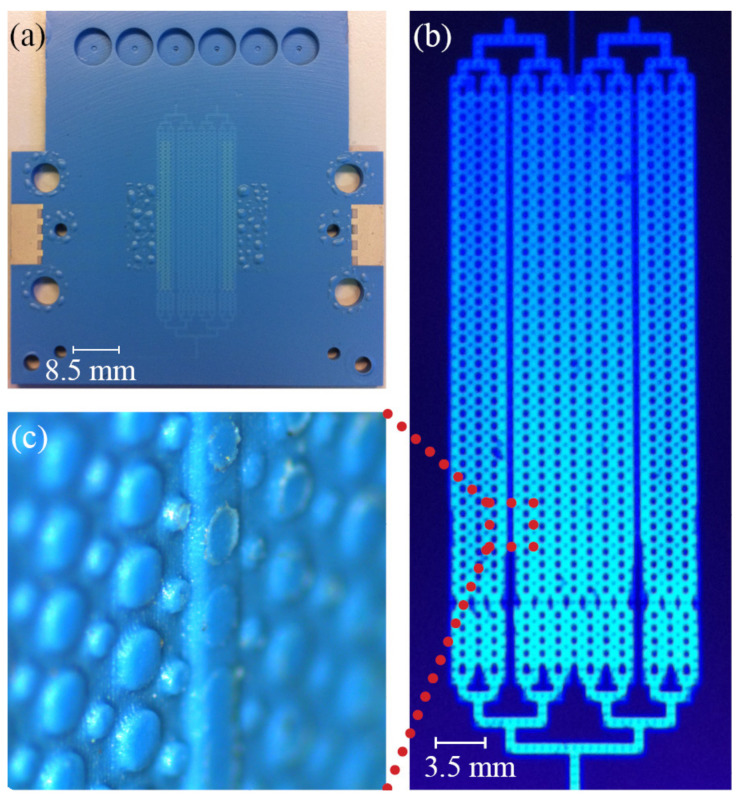
Monolithic ceramic µFFE device design and fabrication. (**a**) Image of the µFFE device. (**b**) Fluorescence image of the µFFE device filled with 4MU solution. (**c**) Open microfluidic chamber image, fabricated using a multilevel design, showing the 10 um deep shallower side channels which separate the 60 um deep electrode chamber from the 60 um deep separation chamber.

The presented microfluidic Free Flow Electrophoresis (µFFE) device is a hybrid design between those proposed by Kobayashy et al. [22] and Raymond et al. [21], with a microfluidic chamber composed of a 60 µm deep separation chamber connected to 50 µm deep electrode chambers by arrays of shallower, 10 µm deep, side channels. The µFFE device was fabricated as a monolithic ceramic device, with integrated, 10 µm thick, Au electrodes and integrated, 20 µm thick, optical windows suitable for fluorescence imaging, as seen in Figure 2b. The integrated optical windows are supported by two pillar matrices, of 500 µm and 250 µm in diameter, both, spaced 1 mm center to center. The sample and buffer microfluidic networks are independent, and the buffer microfluidic network is a manifold that ensures buffer distribution into the separation and electrode chamber in a symmetrical fashion. 

The device was fabricated in low-temperature co-fired ceramics (LTCC) using the fabrication methodology presented in our previous work [27], in which a systematic study on the influence of laser ablation parameters and lamination conditions in the dimensions of microchannels is described. Briefly, since LTCC is a multilayer technology, devices are designed layer by layer. Thereafter, green-state ceramic layers are laser ablated and laminated before the sintering process. Since laser ablation is a direct-write technique, ablated open channel width and depth are determined by the laser energy per unit length used. Our strategy is to control the ablated open microchannels dimension by varying the laser mark speed, which is the velocity with which the laser mirror travels when a line is ablated, while maintaining constant laser power and pulse frequency. Therefore, in order to vary the aspect ratio of the fabricated open microchannels, design strategies must be used. Superposed lines are employed to decrease the aspect ratio of open microchannels, while increasing the open channels aspect ratio can be achieved by ablation of multiple parallel lines. During the lamination process, embedded microchannel dimensions’ decrease with lamination time, reaching a minimum depth and width after 4 min. From this point on, no further microchannel deformation is observed, suggesting a layer-to-layer interpenetration threshold, dependent on temperature and laminating pressure. Therefore, complex device designs can be broken down into multiple LTCC blocks, each fabricated using 4 min as the standard lamination time, which are finally laminated together to obtain the final ceramic device.

**Figure 3:**
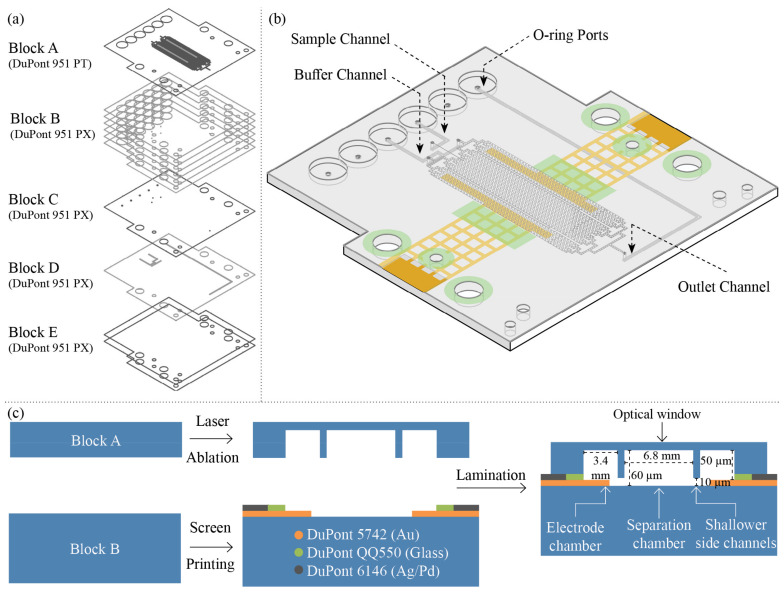
µFFE design and fabrication methodology. (**a**) CAD schematic representation of the layers design arranged in lamination blocks. (**b**) Illustration of the three-dimensional structure of the fabricated monolithic FFE microfluidic device. (**c**) Schematic representation of the cross-section view of the laser ablation process of Block A, screen printing process of Block B, and the final µFFE device (Not to scale).

Using this approach, the presented microfluidic free-flow electrophoresis (µFFE) device was designed layer by layer, using CAD software, as depicted in Figure 3a. DuPont 951 PX (254 µm unfired thickness) layers were used as ceramic substrate in the fabrication of the microfluidic device, except for Block A where a DuPont 951 PT (114 µm unfired thickness) layer was employed. Since LTCC layer transmittance is thickness-dependent [45], in order to maximize transmittance, ceramic optical windows integrated in microfluidic platforms should be as thin as possible. For this reason, the separation and electrode chambers, as well as the shallower side channels, were generated as bas-relief microstructures using two designs, which where ablated into a singular ceramic layer using different laser parameters in a single step, as seen in Figure S1. The separation chamber and the electrodes chambers designed using parallel lines separated by 50 µm, were laser ablated using 100 mm/s laser mark speed, while the shallower side channels were designed using 25 µm parallel line separation and ablated using 250 mm/s laser mark speed. O-ring ports were laser cut using two superposed designs at 25 mm/s mark speed. Using the same methodology, ceramic layer designs of Blocks B to E were laser cut. After laser ablation, the ceramic layers were aligned in aluminum plates and laminated originating blocks A to E, as shown in Figure 3a. Each block was laminated for 5 min. at 70 °C and 30 bar. LTCC co-fireable pastes, Au (DuPont 5742), AgPd (DuPont 6146) and glass encapsulant (DuPont QQ550) where screen printed into Block B as depicted in Figure 3c, and ceramic Blocks where laminated in A to E order for 5 min at 70 °C and 30 bar, before sinterization.

Although the separation and electrode chambers were fabricated using the same laser ablation parameters, the final electrode chamber depth is shallower than the separation chamber, due to the Au electrode, as seen in Figure 3c. Since LTCC technology is derived from thick-film technology, traditional paste deposition techniques, such as screen printing, creates electrodes with thicknesses in the 10 to 100 µm range. The 125 screen printing mesh used creates 10 µm thick Au electrodes.

As seen in Figure 2a, the µFFE device presented bubble-like structural deformation in areas where DuPont QQ550 (glass encapsulant) had been screen printed. DuPont 5742 (Au paste) screen-printed areas will not adhere to other DuPont 951 sheets during lamination. In order to eliminate this problem, DuPont QQ550 (glass encapsulant) was screen printed on top of the DuPont 5742 (Au paste) electrode, as described in Figure 3c. The bubble-like deformations are a consequence of co-firing, to 850 °C, DuPont 951 substrates and DuPont QQ550, which has a firing peak temperature of only 510 to 525 °C. In future designs, in order to avoid such ceramic deformations, DuPont 9615, with a firing peak temperature of 850 °C, should be used for this purpose. This deformation, however, did not have any influence on the µFFE device performance.

### 3.2. Free-Flow Electrophoresis Separation

In free-flow electrophoresis (FFE), the distance migrated by an analyte (*d_x_*) is proportional to the applied field strength (*E*), the residence time of the analytes in the electrical field (*t*), and the analyte electrophoretic mobility (*μ_x_*)

(1) $$d_{x}=E.t.\mu_{x}=SP.\mu_{x}$$

Hence, separation power (*SP*) [25,46] can be used in order to account for the influence of experimental conditions in analytes migration. For the purpose of characterizing the separation capabilities of the µFFE device, *SP* was increased by increasing *E* while maintaining constant *t*. Therefore, a buffer solution (10 mM HEPES, pH 7.0) was injected into the buffer microfluidic network at 40 µL/min while a mixture of fluorescent dyes (0.25 mM of 4MU, 0.25 mM of *Fl*, 0.25 mM of R6G, 0.2% of HPMC in 10 mM HEPES, pH 7.0) was injected into the sample microfluidic network at 0.25 µL/min. A voltage ramp (0–700 V) with 100 V steps was applied at the right electrode, while fluorescence images were registered using the optical detection setup. Analyte residence time of 6 s was experimentally obtained using the described hydrodynamic parameters, while the microfluidic chamber voltage efficiency, modeled as an electrical circuit with 5 resistance connected in series, was calculated to be only 11% due to the high electrical resistance of the side channels array.

**Figure 4:**
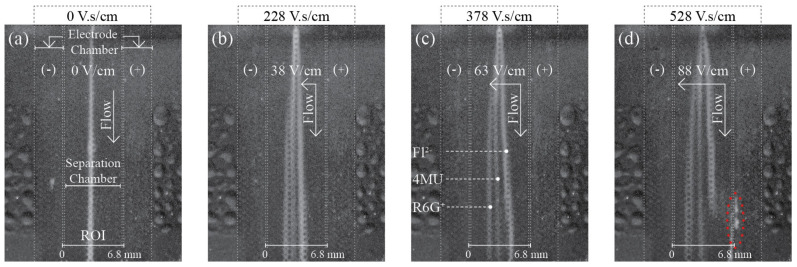
(**a**–**d**) Fluorescence images of the sample mixture separation, using the µFFE device, at increasing separation power and a constant flow velocity of 4.1 mm/s. RGB channel intensities were optimized and converted to grayscale images for printing.

**Figure 5:**
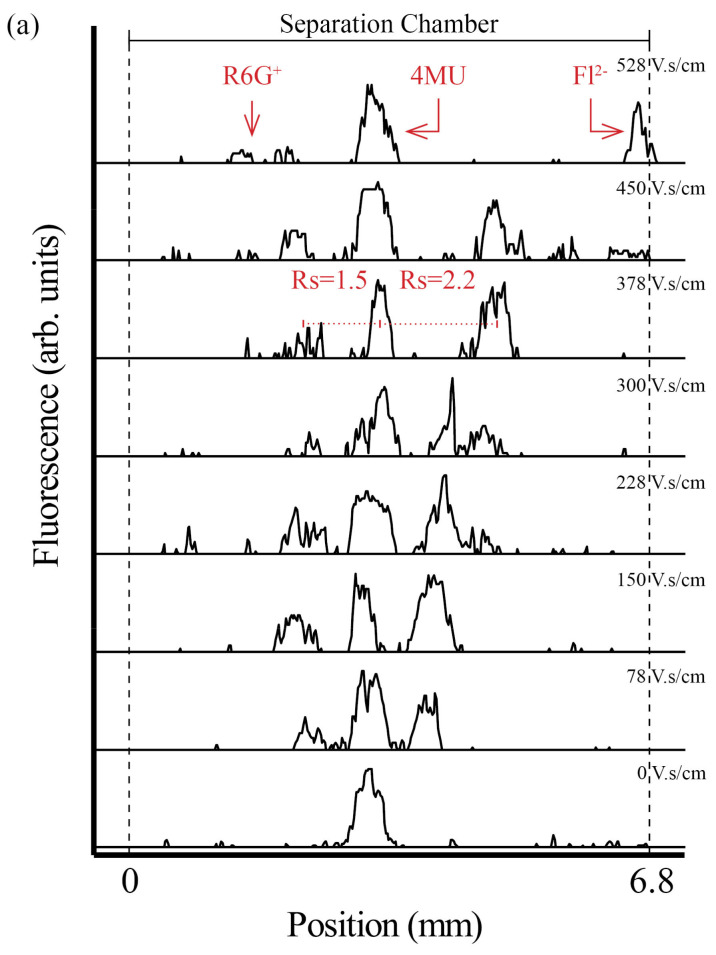

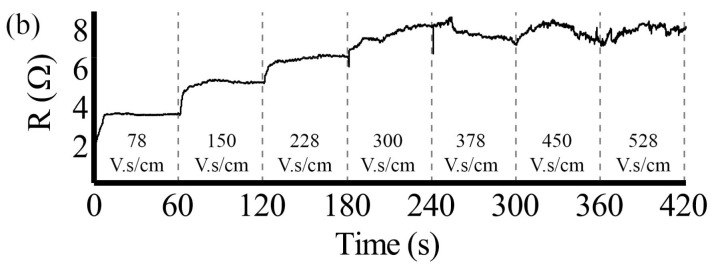
(**a**) Electropherograms, of the sample mixture separation at increasing separation power, recorded at 25 mm from the sample inlet. (**b**) Electrical resistance variation with separation power during the separation process.

As seen in Figure 4, as soon as the electric field was applied, the single sample stream split into multiple streams, and the separation resolution (R) increased with the increase of separation power as expected. At pH 7.0, Fl [47,48] has a −2 charge, and is deflected to the anode while R6G [47] presents a +1 change, and migrates to the cathode. The 4MU [49] is neutral at the used pH and is not affected by the electrical field. The fact that no electrophoretic deflection of 4MU could be observed proves that electroosmotic flow (EOF) can be effectively suppressed in LTCC devices by dynamic coating with HPMC [22], a methodology previously described for EOF suppression in glass devices. Baseline separation (R > 1.5) was obtained for all analytes, at 378 V.s/cm, as depicted in Figure 5a. 

For SP higher than 378 V.s/cm, the Fl linear migration was disrupted and a fluorescent band appeared in the separation chamber and side channel interface, as depicted in Figure 4d. According to Equation (1), this phenomenon can be explained by an increase in the analyte electrophoretic mobility, which suggests a variation of the chemical environment in that region.

**Figure 6:**
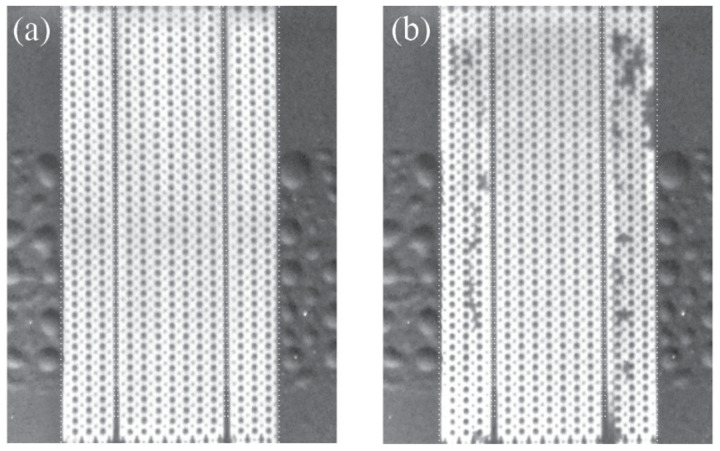
Fluorescence images of the microfluidic chamber filled with the sample mixture (0.25 mM of 4MU, 0.25 mM of Fl, 0.25 mM of R6G, 0.2% of HPMC in 10 mM HEPES, pH 7.0), (**a**) before the separation process and (**b**) after the separation process. Gas bubbles are identifiable as non-fluorescent regions inside the electrode chamber.

As shown in Figure 5b, the µFFE device electrical resistance increased with increasing SP. This is the consequence of water electrolysis gaseous products accumulation, revealing the limited capacity of the µFFE device design to flush increasing amounts of O_2_ and H_2_ generated at increasing SP. As a consequence of the high area to volume ratio at the microscales, gas bubbles are extremely difficult to eliminate in microfluidic systems and will not be flushed away by continuous injection of aqueous solution. Therefore, to verify the presence of gas bubbles in the separation chamber, the microfluidic chamber was filled, before and after each experiment, with the sample solution. The results, depicted in Figure 6, show that no bubbles generated from water electrolysis entered the separation chamber during the separation process, proving the effectiveness of the side channels high-hydrodynamic-resistance design under the experiential conditions used.

### 3.3. Spatial-Temporal Characterization of pH during the Separation Process

As described previously, for SP higher than 378 V.s/cm, the Fl band migration was disrupted and a fluorescent band appeared at the interface of the separation chamber with the side channels. To determine whether this phenomenon could be the consequence of local pH variations in separation conditions, as suggested by Equation (1), 4MU solution (0.25 mM 4MU, 0.2% of HPMC in 10 mM HEPES, pH 7.0) was used as a pH probe. Using the same electrical and hydrodynamic parameters as in the separation experiments, 4MU solution was injected, through both the sample and buffer microfluidic networks, into the separation chamber. 

**Figure 7:**
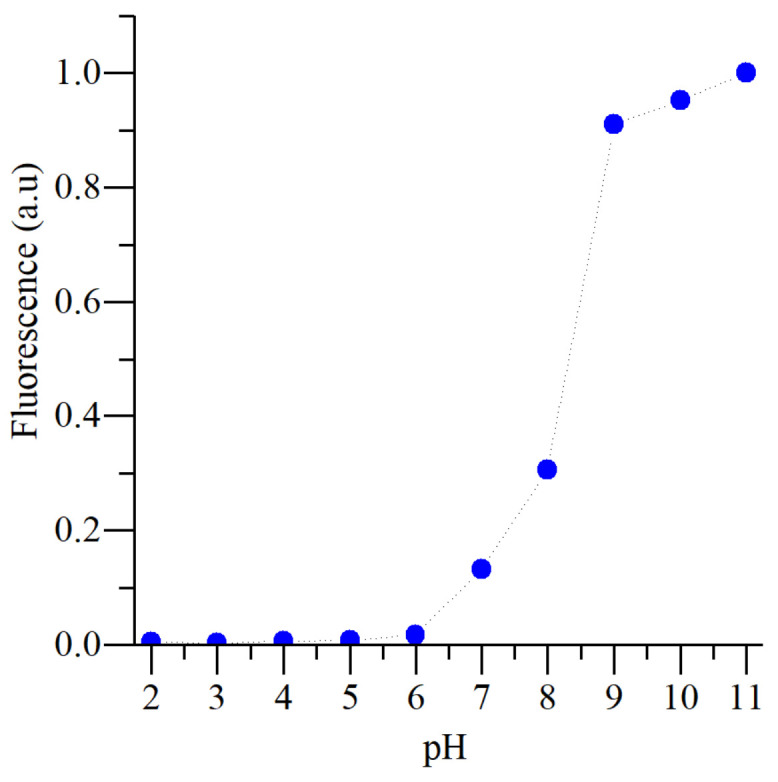
Plot of fluorescence intensity variation with pH of 4MU (0.1 mM), for λ_excitation_ = 365 nm and λ_emission_ = 450 nm, obtained using a spectrofluorometer.

The fluorescence intensity of 4MU [49] is pH-dependent, as depicted in Figure 7, having minimum fluorescence in an acidic medium and reaching maximum intensity above pH 10.0 when the fluorescence is approximately 10 times as intense as at pH 7.0. Moreover, no 4MU fluorescence quenching effect occurs in the presence of O_2_ [50], making it an ideal probe for pH spatial-temporal characterization of electrophoresis processes, where O_2_ is generated in situ due to water electrolysis. 

**Figure 8:**
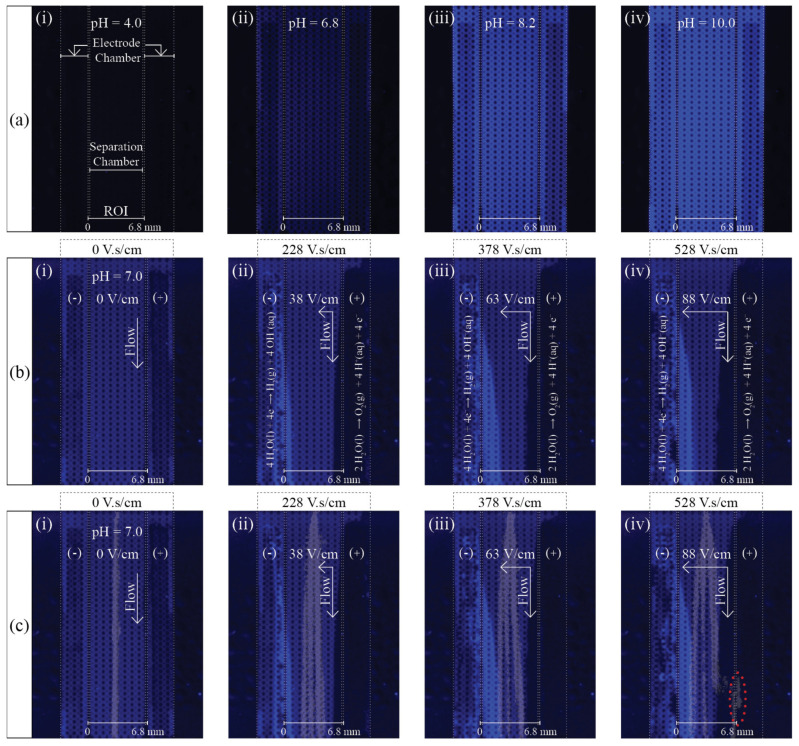
Spatial-temporal characterization of the microfluidic chamber local pH using fluorescence imaging of 4MU solution (0.25 mM 4MU, 0.2% of HPMC in 10 mM HEPES, pH 7.0). (**a**) Fluorescence images of 4MU solution at different pH. (**b**) Fluorescence images of 4MU solution in separation conditions at increasing separation power and constant flow velocity of 4.1 mm/s. (**c**) Superposed fluorescence data from 4MU solution in separation conditions, depicted in Figure 8b, and the sample mixture separation, depicted in Figure 4.

**Figure 9:**
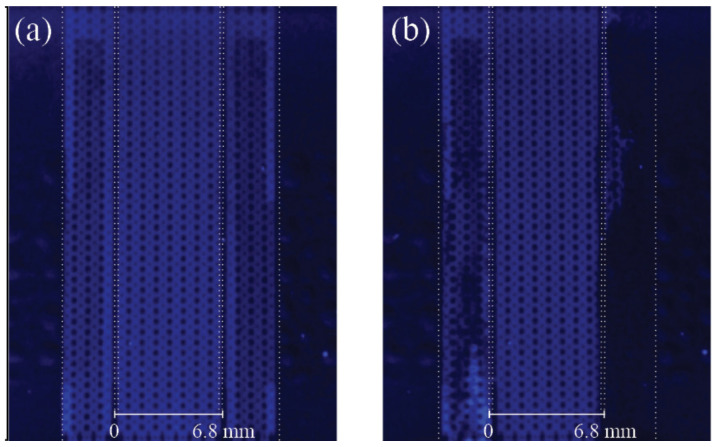
Fluorescence images of the microfluidic chamber filled with 4MU solution (0.25 mM 4MU, 0.2% of HPMC in 10 mM HEPES, pH 7.0), (**a**) before the experiment and (**b**) after the experiment.

As shown in Figure 8a, the high dynamic fluorescence intensity range of 4MU allowed pH maps to be obtained using our simple and low-cost fluorescence optical system composed of a consumer CCD camera and a consumer UV light lamp. HEPES buffer, used in this work at pH 7.0, has a work pH range from 2.5 to 3.5 and 6.8 to 8.2. Therefore, any regions where the buffer capacity is disrupted should be easily identified. Non-fluorescence areas characterize acidic regions and high-intensity blue areas depict alkaline regions. Non-fluorescence regions may also illustrate the presence of gas bubbles. As seen in Figure 8b, as soon as the electric field was applied a bright blue region appeared in the cathode electrode chamber. The bright blue area increased with increasing SP and the region expanded into the separation chamber when the SP exceeded 228 V.s/cm. The bright blue area represents a region where the buffer’s capacity to maintain a stable chemical environment was exceeded, as a consequence of OH^−^ produced by water electrolysis. Non-fluorescent areas, located insight the bright blue region, correspond to H_2_ gas bubbles produced in the same reaction. Since the amount of OH^−^ also increases with increasing SP, the area becomes a high-pH region. Likewise, the buffer’s capacity is exceeded in the anode, where the generation of H^+^, by water electrolysis, leads to the generation of low-pH regions. Since, as seen in Figure 6 and Figure 9, no bubble generated from water electrolysis entered the separation chamber in any of the experiments, the non-fluorescent area in the anode proximity corresponds to a low-pH area. The acidic area increased with increasing SP and expanded into the separation chamber when SP reached 228 V.s/cm. Therefore, the effective separation area of the µFFE device is not defined by the separation chamber geometry but rather by the region where the buffer maintains constant pH conditions. 

In Figure 8c, the experimental data described in Figure 8b and in Figure 4 have been superposed in order to compare both experiments. At 378 V.s/cm the Fl band is at the frontier of the buffered regions while R6G is already inside the basic pH region. The transition from a stable pH environment to a basic pH region has no effect on the R6G stream flow paths. The R6G electrophoretic mobility [47] is pH independent as a result of R6G not having a pKa and, therefore, not undergoing acid-base equilibrium. On the other hand, for SP higher than 378 V.s/cm, the Fl stream enters the acidic region, therefore, undergoes protonation [48], as described in Equation (2).

(2) $$FlH_{3}^{+}\overset{PK_{1}=2.08}{\Leftrightarrow }FlH_{2}\overset{PK_{2}=4.03}{\Leftrightarrow }FlH^{-}\overset{PK_{3}=6.43}{\Leftrightarrow }Fl^{2-}$$

Although *Fl* presents the highest electrophoretic mobility when in the *Fl*^2−^ form [47,48], the existence of a fluorescent *Fl* band at the interface of the separation chamber with the side channels structure, suggests that a significant increase in electrophoretic mobility occurs in the acidic region. The mechanism responsible for this phenomenon is unclear and should be further investigated. Nevertheless, the obtained pH maps suggest a clear relationship between the *Fl* stream flow paths and local pH, allowing a comprehensive visual description of the disruption of the buffer capacity to maintain a stable chemical environment due to chemical contamination by water electrolysis products (H^+^ and OH^−^).

## 4. Conclusions

A monolithic microfluidic Free Flow Electrophoresis device has been fabricated, for the first time to our knowledge, using LTCC technology. The µFFE device presents a multilevel microfluidic chamber design with integrated gold electrodes, as well as an integrated transparent ceramic optical window suitable for fluorescence imaging. The described fabrication procedure allows the fabrication of hermetically sealed microfluidic devices with homogeneous surface chemistry and physical properties.

The µFFE device allowed baseline resolution, for all peaks, of a mixture Fluorescein, Rhodamine 6G, and 4-Methylumbelliferone, at pH 7.0, using a separation power of 378 V.s/cm. Fluorescence imaging of 4MU, used as pH indicator, allowed spatial-temporal characterization of local pH variations during the separation process. Until now, µFFE designs focused on the development of strategies to prevent physical products (gas bubbles) of water electrolysis from entering the separation chamber and disrupting the separation flow paths. Nonetheless, in the separation conditions used, the separation-limiting factor was not the elimination of physical products of water electrolysis but, rather, the contamination of the separation buffer with the water electrolysis’s chemical products (H^+^ and OH^−^), which effectively disrupted the buffer capacity to maintain a stable chemical environment, consequently decreasing the effective separation area. Although this phenomenon has been previously hypothesized [17] this is, as far as we know, the first time experimental data was obtained allowing a comprehensive description of spatial-temporal variation of local pH during the separation process.

Moreover, the obtained results show that fluorescence imaging is a powerful tool for microfluidics applications, opening the possibility of a comprehensive visual description of chemical processes in static or flow conditions using simple and low-cost experimental setups.

## Data Availability

Not applicable.

## Supplementary Materials

The following supporting information can be downloaded at: https://www.mdpi.com/article/10.3390/mi13071023/s1.
