# Supplementary material for: Fluorescence Imaging Characterization of the Separation Process in a Monolithic Microfluidic Free-Flow Electrophoresis Device Fabricated Using Low-Temperature Co-Fired Ceramics
Source: Micromachines (Basel). 2022 Jun 28;13(7):1023. doi: 10.3390/mi13071023 (PMC9324176; doi:10.3390/mi13071023)
Supplement: Supplementary file 1 [file micromachines-13-01023-s001.zip › micromachines-1771803-supplementary.pdf]

Supplementary Materials

Fluorescence imaging characterization of the separation process in a monolithic microfluidic Free Flow Electrophoresis device fabricated using Low Temperature Co-Fired Ceramics.

Pedro Couceiro and Julián Alonso-Chamarro*

Sensors & Biosensors Group, Department of Chemistry, Autonomous University of Barcelona, Edifici Cn, 08193 Bellaterra, Catalonia, Spain

Corresponding Author email: Julian.Alonso@uab.es

ORCID:

Julián Alonso-Chamarro : 0000-0002-6804-6027

Pedro Couceiro : 0000-0001-5539-2441

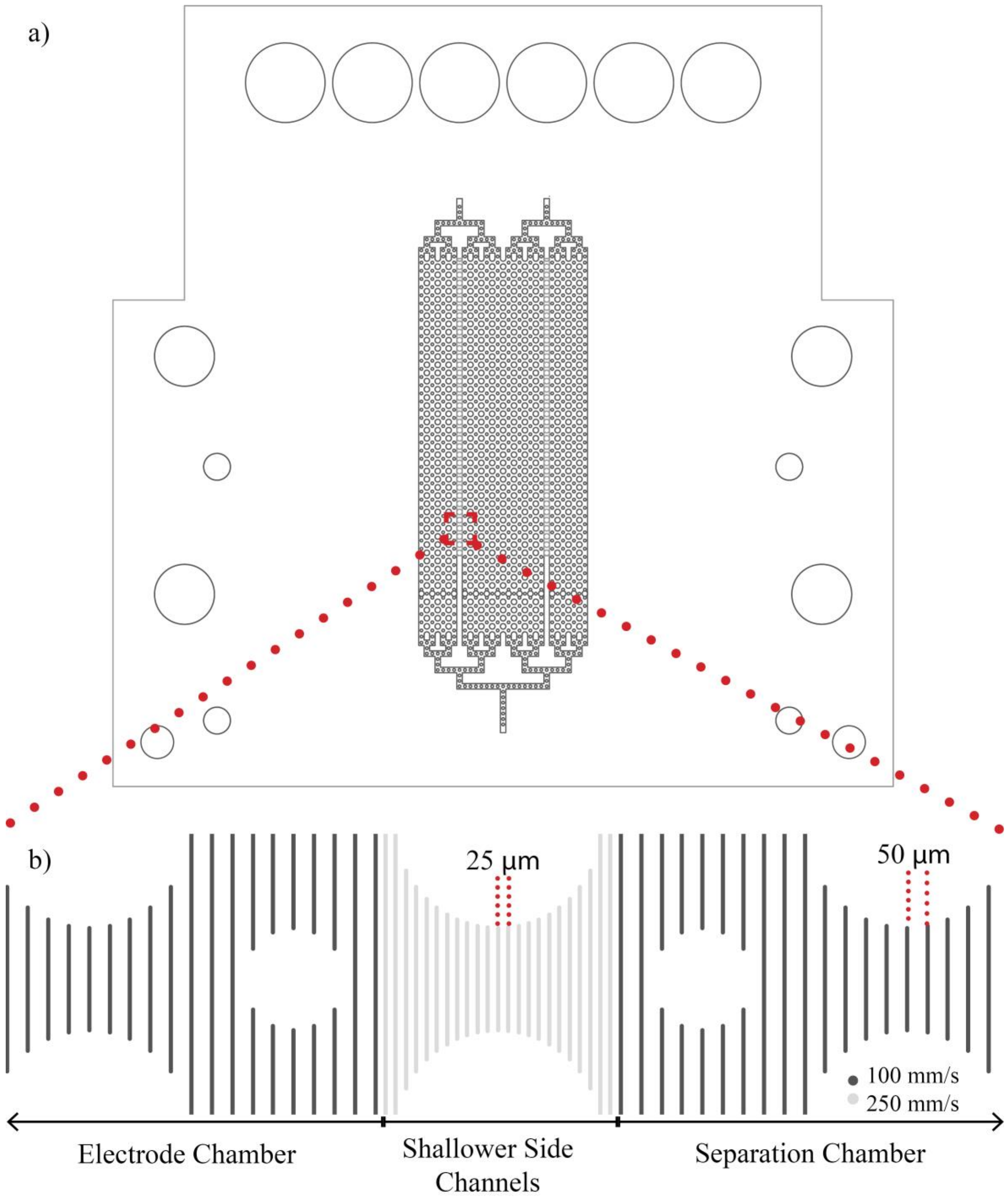

Figure S1. Block A design and laser ablation conditions. a) Schematic representation of Block A CAD design. b) CAD design detail, with laser ablation lines and laser ablation process conditions, of the Electrode Chamber, Shallower Side channels and Separation Chamber. Areas with no ablation lines originate pillar while areas with ablation lines originate microchannels.
